# Investigation of an Electrochromic Device Based on Ammonium Metatungstate-Iron (II) Chloride Electrochromic Liquid

**DOI:** 10.3390/mi13081345

**Published:** 2022-08-19

**Authors:** Sifan Kong, Guanguang Zhang, Muyun Li, Rihui Yao, Chenxiao Guo, Honglong Ning, Jianzhi Zhang, Ruiqiang Tao, Haoyang Yan, Xubing Lu

**Affiliations:** 1 School of Software, South China Normal University, Foshan 528225, China; 2 Institute of Polymer Optoelectronic Materials and Devices, State Key Laboratory of Luminescent Materials and Devices, South China University of Technology, Guangzhou 510640, China; 3 School of Physics & Photoelectric Engineering, Guangdong University of Technology, Guangzhou 510650, China; 4 Institute for Advanced Materials, South China Academy of Advanced Optoelectronics, South China Normal University, Guangzhou 510006, China

**Keywords:** electrochromism, no-film device, ammonium metatungstate, iron (II) chloride, lifetime, current density

## Abstract

Even though electrochromism has been around for more than 50 years, it still has several issues. Multi-layered films, high manufacturing costs, and a short lifetime are present in existing electrochromic devices. We demonstrate a unique high-performance device with a basic structure and no solid electrochromic sheets in this work. In this device, the electrolyte layer is also avoided. The device uses an electrochromic solution prepared from a mixture of ammonium metatungstate and iron (II) chloride solution as a functional layer with reversible redox properties. The tungstate ions on the electrode surface are reduced when the device is colored, and the Fe^2+^ on the electrode surface is oxidized on another electrode surface. The generated Fe^3+^ in the mixed functional layer oxidizes the previously reduced tungstate ions as the device fades. We determined the ΔT (transmittance modulation) and response time among ammonium metatungstate ratios, iron (II) chloride ratios, and driven current density using DOE (design of experiment) trials. Using 0.175 mol/L ammonium metatungstate and 0.30 mol/L iron (II) chloride, a device with outstanding ΔT (more than 57% at 700 nm), a short response time (less than 10 s), and high coloring efficiency (160.04 cm^2^/C at 700 nm) is demonstrated.

## 1. Introduction

The design of energy-efficient houses has become a hot research topic because buildings consume roughly one-third of the world’s total energy usage [1,2,3]. By adjusting the visible and solar energy input through voltage and current application, electrochromic smart windows can be used to reduce energy consumption for room cooling [4,5,6]. Electrochromism, defined as a material’s ability to dynamically vary its optical characteristics in response to an applied voltage or current stimulation, is expected to have a wide range of applications [7,8]. Inorganic electrochromic compounds are gaining popularity due to their environmental friendliness and stability [9]. Cathodic electrochromic materials and anodic electrochromic materials are the two types of inorganic electrochromic materials that are extensively employed. Cathodic electrochromic materials include WO_3_, MoO_3_, Nb_2_O_5_, TiO_2_, Ta_2_O_5_, and their derivatives or mixtures. In addition, other materials, including NiO, IrO_2_, V_2_O_5_, and Prussian blue analogs, are also used as anodic electrochromic materials [10,11]. Tungsten trioxide (WO_3_) is the most widely studied and used electrochromic material. It has been applied to various optical devices. Due to its attractive qualities of high coloring efficiency, good optical modulation, and high chemical stability in the field of electrochromism [12,13], WO_3_ has been investigated for many years. The emergence of electrochromic (EC) smart windows has sparked renewed interest in WO_3_, which may help in reducing energy consumption in buildings [14,15,16]. According to architectural simulations, utilizing smart windows instead of standard static windows for space cooling can save up to 40–50% of energy in fairly warm climates [17]. WO_3_-based EC smart windows made by magnetron sputtering have been commercially accessible as the only EC smart window on the market for more than 10 years [18]. However, several challenges, such as coloring efficiency [10,19,20,21], are preventing magnetron-sputtered electrochromic smart window performance from being improved. Meanwhile, due to poor EC performance and unreasonably high prices of vacuum equipment and processing, this type of EC smart window has not been extensively implemented [22]. Solution-processed EC smart windows have high contrast ratios (>90%) [23], quick reactions (less than 1 s) [22], and good coloring efficiency (141.5 cm^2^/C) [24]. Their cycle life and coloring efficiency can still be improved, resulting in a stronger impact on practical applications [20,25,26]. There has been a lot of research into existing all-solid-state electrochromic devices. Pure liquid electrochromic devices, on the other hand, are a growing area of research. Tungstate has high solubility and electrochromic activity, making it a viable candidate for liquid electrochromic devices [27]. Meanwhile, a liquid electrochromic device based on organic electrochromic materials has been researched [28]. Liquid electrochromic devices based on inorganics are a direction of research.

The main electrochromic material used in the device reported in this study is an inorganic iron–tungsten salt. In conjunction with a three-layer electrode/electrochromic liquid/electrode non-polar device, an in situ electron donor–receiver separation/compounding strategy is proposed that entails a simple process and does not involve an expensive gas environment or complex operation. The process only requires the injection of the electrolyte between two indium tin oxide glass pieces. Benefitting from this process and simple craft, the cost of this device is dramatically lower than that of the current commercial device. Therefore, it makes sense to research the device in greater depth. We demonstrate that the device shows non-destructive electrode color change and fading, with a cycle time of more than 100 times and a response time of less than 10 s, surpassing the physical limit of the coloring efficiency of the traditional film layer structure. An experiment on the mixed functional layer was conducted following the directions of the software Minitab. The various factors and levels considered in this work include the concentration of ammonium metatungstate, current density, and iron (II) chloride concentration. Using the constant current approach, the microscopic mechanism of the electrochromic process in the mixed functional layer was investigated.

## 2. Materials and Methods

### 2.1. Solution Preparation

**Figure 1:**
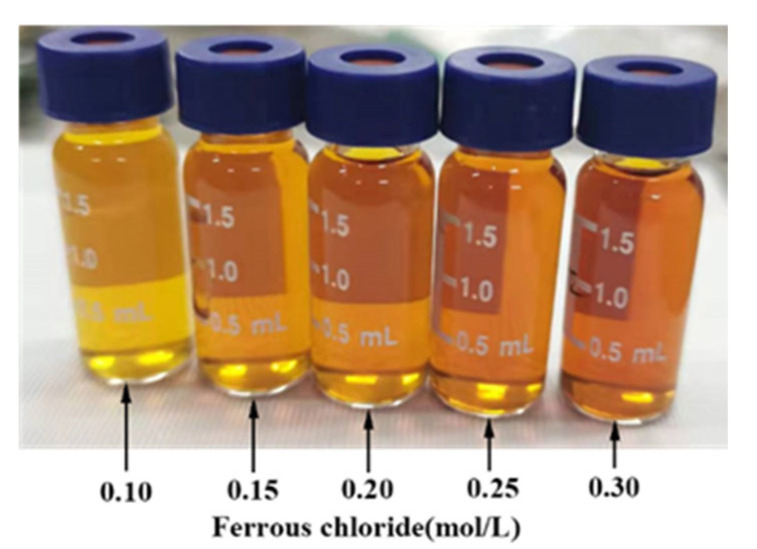
Solutions of progressively increasing concentration of iron (II) chloride from left to right with a concentration of 0.175 mol/L ammonium metatungstate.

First, 5 mL of deionized water and both ammonium metatungstate (AMT, (NH_4_)_6_H_2_W_12_O_40_·xH_2_O, 99.5%, Guangzhou Chemical Reagent Factory, Guangzhou, China) and iron (II) chloride (FeCl_2_·4H_2_O, Macklin Biochemical Co., Ltd., Shanghai, China) were added to a beaker to prepare a mixture of ammonium metatungstate and iron (II) chloride. Then, solutions with various concentrations of ammonium metatungstate in the solution were made: 0.100 mol/L, 0.125 mol/L, 0.150 mol/L, 0.175 mol/L, and 0.200 mol/L. The concentrations of iron (II) chloride were 0.10 mol/L, 0.15 mol/L, 0.20 mol/L, 0.25 mol/L, and 0.30 mol/L. The precursor solution was produced by sonicating the solutions. Referring to Table 1, the experiment samples are sorted into 25 groups by Taguchi design. The color of the solution changes from yellow to dark brown as the iron (II) chloride concentration rises. Figure 1 shows one example (The 25 samples’ UV-VIS can be found in the Supplementary Materials).

### 2.2. Electrochromic Device Preparation

**Figure 2:**
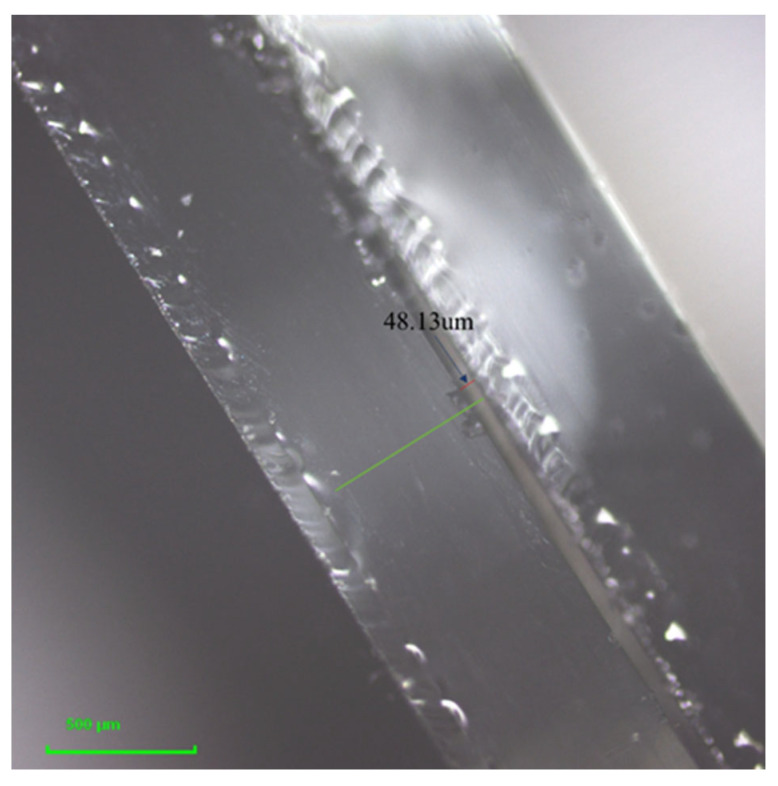
The confocal laser scanning microscope (CLSM) image of the device sample.

Indium tin oxide (ITO) glass substrates (4 × 4 cm^2^) were ultrasonically cleaned with deionized water for 15 min and subsequently with anhydrous ethanol for 15 min. Afterwards, conductive gel with a thickness of 50 μm on the ITO glass was used to bond the two pieces of glass. Finally, using the indium tin oxide (ITO) flat glass substrate (4 × 4 cm^2^) with thin layers (2 × 3 cm^2^), all solutions were injected into the gap between two pieces of ITO glass using the capillary method. The mixed functional layer had a thickness of roughly 48 μm, as shown in Figure 2.

### 2.3. Performance Characteristics

The current for the electrochromic test was provided by an electrochemical workstation (CH Instruments CHI660E, CH Instruments, Shanghai, China), and the current densities were obtained by calculating the input constant current and layer area as 1 A/m^2^, 2 A/m^2^, 3 A/m^2^, 4 A/m^2^, and 5 A/m^2^. Using the Taguchi design principle, different metatungstate concentrations (DOE experiment), iron (II) chloride concentrations, and current densities were divided into 25 groups.

A microspectrometer (Morpho PG2000, Morpho, Shanghai, China) was used to record variations in transmittance over time, with air serving as a blank. The ΔT of the test devices was observed using a constant current input for 30 s. The change in transmittance of the devices was observed using a constant current input. From the preceding trials, the highest-performing device was chosen to test the device longevity with sets of 30 s of constant current and 30 s of power off for a total of 100 cycles.

## 3. Results and Discussion

### 3.1. Electrochromic Principle

The electrochromic principle of the experimental mixed functional layer is mainly the oxidation of tungstate [29,30,31].

(1) $${\left[H_{2}W_{12}^{\mathrm{VI}}O_{40}\right]}^{6-}{+(2a+b)e}^{-}\to {{[H}_{2}W_{a}^{\mathrm{IV}}W_{b}^{V}W_{12-\left(a+b\right)}^{\mathrm{VI}}O_{40}]}^{\left(6+2a+b\right)-}\;(\mathrm{Coloring})$$

(2) $$\left(2a+b){\mathrm{Fe}}^{2+}\to {(2a+b)\mathrm{Fe}}^{3+}+{(2a+b)e}^{-}\;(\mathrm{Coloring}\right)$$

(3) $${{[H}_{2}W_{a}^{\mathrm{IV}}W_{b}^{V}W_{12-\left(a+b\right)}^{\mathrm{VI}}O_{40}]}^{\left(6+2a+b\right)-}{+(2a+b)\mathrm{Fe}}^{3+}\to {\left[H_{2}W_{12}^{\mathrm{VI}}O_{40}\right]}^{6-}{+(2a+b)\mathrm{Fe}}^{2+}\;\;\left(\mathrm{Bleaching}\right)$$

**Figure 3:**
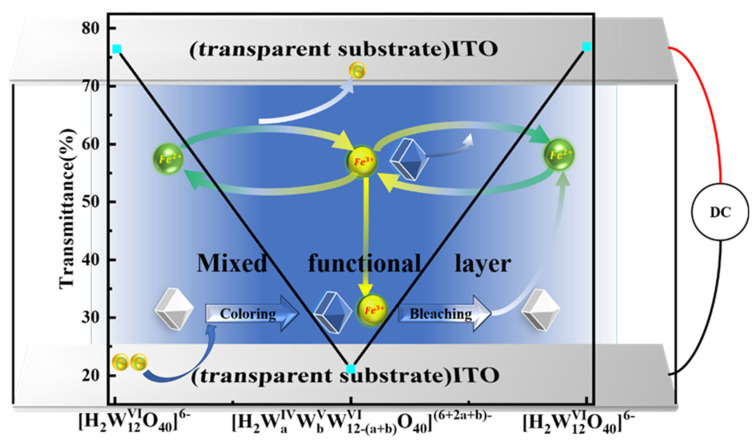
Reaction diagram of the mixed functional layer.

Figure 3 shows a complex electrochemical reaction in which W^IV^, W^V^, and W^VI^ are present simultaneously [29,30,31]. When energization begins, the metatungstate on the cathode surface is decreased, the iron (II) ions are oxidized to iron (III) ions on the anode surface, and the mixed functional layer changes from light yellow to blue. The leftover iron (III) ions remain in the solution after energization stops. The upper limit of the number of molecules containing reduced tungsten atoms in the solution at the time of energization is determined by the concentration of ammonium metatungstate, which affects the upper limit of ΔT and the device’s response time, whereas the current density affects the number of molecules of reduced tungsten atoms and Fe^3+^ in the solution at the time of energization and the device’s response time. The concentration of iron (III) chloride can affect the amount of Fe^3+^ in the solution at the time of energization and the response time of the device. The concentration of iron (II) chloride can affect the amount of Fe^3+^ in the solution after a power failure. The aim of setting up 25 sets of experiments was to find the device with the best performance. The amount of Fe^3+^ in the solution at the moment of energization and the device’s response time can be affected by the initial iron (II) chloride concentration. After a power outage, the concentration of iron (II) chloride in the solution can impact the amount of Fe^3+^ in the solution. The goal of running 25 sets of experiments was to determine the device that performed the best.

### 3.2. Transmission Modulation Amplitude

**Figure 4:**
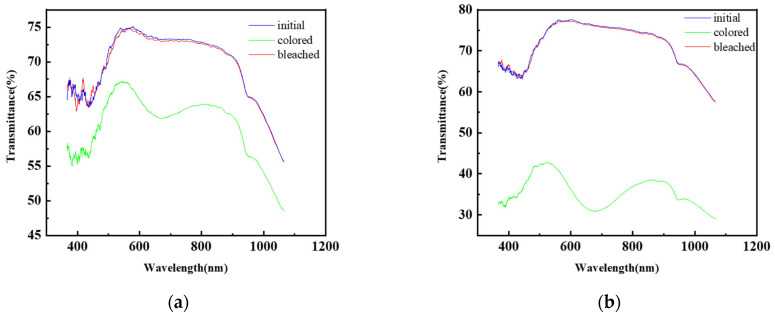
The optical transmittance spectra of the two different samples of the mixed functional layer in the initial, bleached, and colored state: (**a**) 0.175 mol/L ammonium metatungstate × 0.25 mol/L iron (II) chloride group at a current density of 1 A/m^2^; (**b**) 0.175 mol/L ammonium metatungstate × 0.20 mol/L iron (II) chloride group at a current density of 5 A/m^2^.

Figure 4a,b show that the main range for the optical transmittance spectra of the mixed functional layer in the initial state, colored state, and bleached state is 650–750 nm. In order to compare different experimental groups, we select 700 nm as the reference wavelength.

The transmittance modulation ability (∆*T*) can be defined at a 700 nm wavelength by the following formula:

(4) $$\Delta T=\left|T_{c}-T_{b}\right|$$

Comparing the degree of transmittance during the switching process can provide ∆*T*. ∆*T* is used to measure the optical modulation ability of the mixed functional layer [32,33]. The larger the ∆*T*, the better the optical modulation ability of the mixed functional layer. Current density, ammonium metatungstate concentration, and iron (II) chloride concentration are all parameters that influence ∆*T* in the mixed functional layer. The effects of ammonium metatungstate concentration, iron (II) chloride concentration, and current density on ∆*T* were investigated in 25 tests.

**Figure 5:**
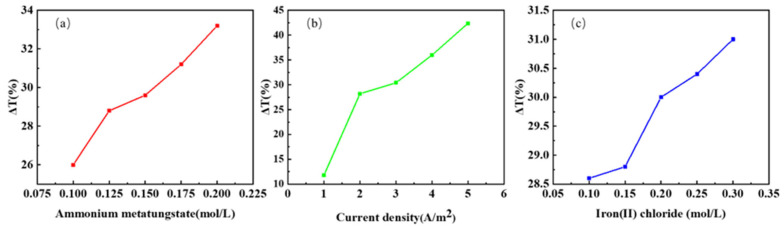
The mean main effect diagrams of different factors for ΔT: (**a**) ammonium metatungstate; (**b**) current density; (**c**) iron (II) chloride.

Figure 5 shows the main effect diagrams for various factors obtained from the 25 sets of data through optimization.

Figure 5a–c show the main effects on ∆*T*, demonstrating that ammonium metatungstate, iron chloride, and current density are all significant influences that are proportional to ∆*T*. Regression analysis can be used to generate the regression equation for varying current densities by using the current density as a category prediction model. The regression equation is as follows:

(5) $$C=5\;A/m^{2}:\Delta Transmittance=29.76+67.2C_{A}+12.80C_{F}$$

where *C*, Δ*T*, *C_A_*, and *C_F_* represent the current density, the transmittance modulation ability, and concentrations of ammonium metatungstate and iron (II) chloride, respectively. R-sp (95.46%) means that the regression equation fits well. This regression equation shows that ammonium metatungstate has a greater effect on the transmission modulation amplitude than iron (II) chloride, indicating that ammonium metatungstate is the most important component for determining Δ*T* in the experiment. Following the end of the 25-group experiment, concentrations of 0.225 mol/L ammonium metatungstate and 0.30 mol/L iron (II) chloride were introduced as a supplementary experimental group (see Supplementary Materials), and the conclusion is provided in the last part. The conductivity was calculated using the following equation:

(6) $$\sigma =\frac{d}{R_{b}S}$$

Here, *d* (cm) is the thickness, *S* (cm^2^) is the electrode–electrolyte contact area (6 cm^2^), and *R_b_* is the volume resistance in ohms [34]. This formula leads to the resistance of the solution:

(7) $$R=\frac{d}{\sigma S}$$

Usually, the ionic conductivity (*σ*) of an electrolyte can be described by the following equation:

(8) σ(T)=Σ n×q×μ

**Figure 6:**
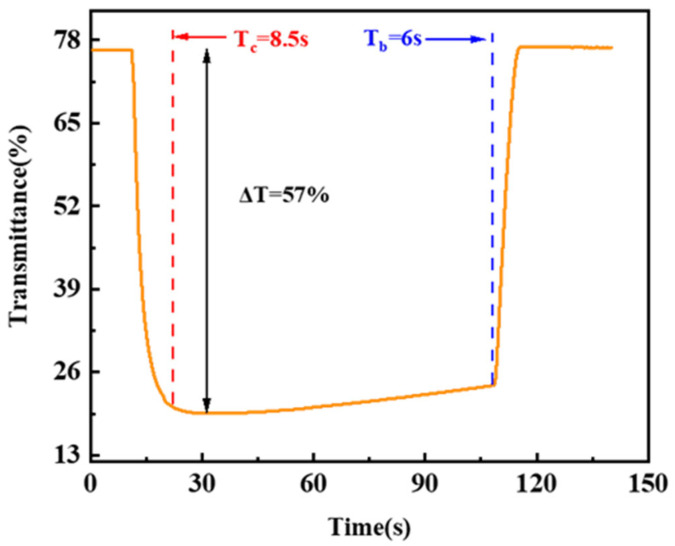
The transmittance–time diagram at 700 nm for the 0.175 mol/L ammonium metatungstate × 0.30 mol/L iron (II) chloride group at a current density of 6 A/m^2^.

Here, *n* is the number of charge carriers, *q* is the charge on the charge carrier, and *μ* is the mobility of the charge carrier. If one of the two individual carriers is being analyzed, the ionic conductivity *σ* increases as the concentration of that ion in the solution increases, given that both *q* and *u* are the same [35]. As a result of the aforementioned derivation, the concentrations of ammonium metatungstate and iron (II) chloride in the solution increase, and the resistance of the device decreases. However, prolonged high-voltage exposure to the mixed functional layer can cause ammonium metatungstate crystallization and the loss of electrochromic characteristics. The high voltage causes the crystallization of W^4+^ in the solution. When this phenomenon occurs, due to the dense crystal structure, the crystallized W^4+^ in the solution cannot quickly release/insert ions [36]. Meanwhile, in contrast to the state of the free ion in the solution, crystallized W^4+^ in the solution is too tough to be oxidized by Fe^3+^ in the solution, drastically reducing the performance of the device by affecting the initial Δ*T* and other parameters of the mixed functional layer. As the ions in the solution increase, according to Formulas (6) and (7), the resistance of the mixed functional layers will decrease, which will decrease the voltage of the device when the currents are the same, so the limit of the current density can increase (More content about this principle can be found in the Supplementary Materials). The current density is thus limited by the concentrations of ammonium metatungstate and iron (II) chloride, which cannot be consistently increased, and the upper limit increases with solution concentration, resulting in a better transmission modulation amplitude at higher current densities. This argument is supported by Figure 6, which shows that under these conditions, Δ*T* can reach 57%, much higher than the best values in the design experiments. In summary, increasing the concentrations of ammonium metatungstate and iron (II) chloride can increase the upper limit of the current density. It performs well when compared to several standard procedures and commercial techniques on the market, as shown in Table 2.

### 3.3. Response Time

**Figure 7:**
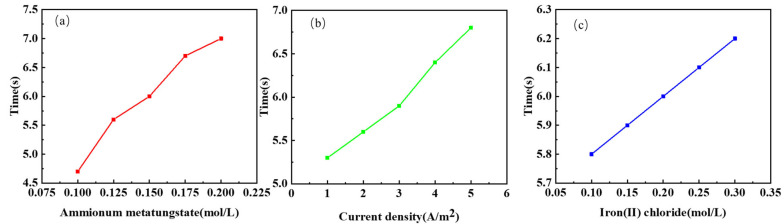
The mean main effect diagrams of different factors for the response time: (**a**) ammonium metatungstate; (**b**) current density; (**c**) iron (II) chloride.

Figure 7a–c show the main effects plot of the response time, which shows that the response time is related to the ammonium metatungstate concentration, current density, and iron (II) chloride concentration. The response time increases with increasing current density, further demonstrating that, in the experimental group, the current density contributes more strongly to the amplitude of the transmission modulation than it does to reducing the response time, making it impossible to reduce the response time by increasing the current density. Meanwhile, the response time in all experiments was less than 10 s. Besides, compared with some work, this work’s performance of response time is well (see Table 3).

### 3.4. Coloring Efficiency

The ratio between the change in optical density (*OD*) and the value of charge per unit area (*Q*) in the mixed functional layer is precisely defined as coloring efficiency (*CE*), which is statistically calculated as [43]:

(9) $$CE=\frac{\Delta \left(OD\right)}{\Delta Q}=\frac{log\left(\frac{T_{b}}{T_{c}}\right)}{\Delta Q}$$

**Figure 8:**
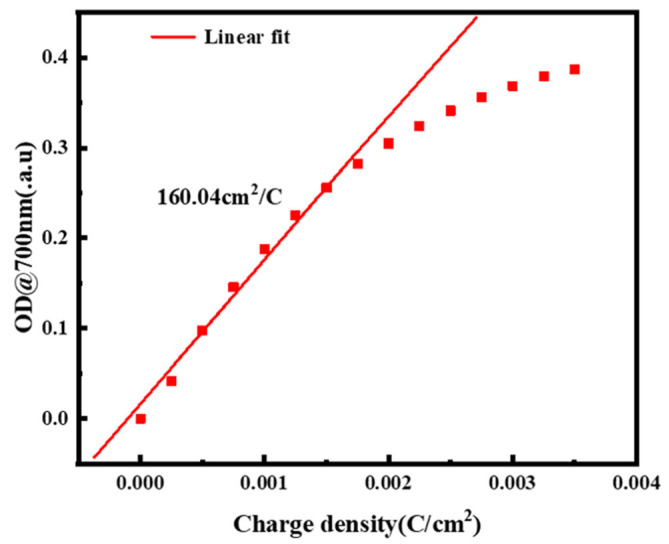
The coloring efficiency diagram at 700 nm for ammonium metatungstate concentration of 0.175 mol/L and iron (II) chloride concentration of 0.20 mol/L at a current density of 5 A/m^2^: scatter plot of coloring efficiency.

Coloring efficiency is an important factor for measuring the energy-related efficiency of electrochromic materials and devices. Figure 8 shows that the device has excellent coloring efficiency. We infer that the device’s coloring efficiency is 160.04 cm^2^/C, which represents extremely high energy efficiency. The performance is far superior compared to some conventional electrochromic devices, refer to Table 4. This is because liquid materials have a large specific surface area and do not suffer from the material build-up issues that traditional material films do.

**Figure 9:**
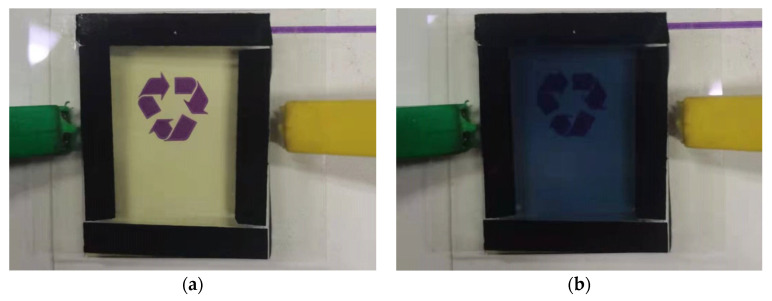
(**a**) Optimized experimental group in the colored state; (**b**) optimized experimental group in the faded state.

After the optimization of the DOE experiment, we obtained the optimized experimental group (0.175 mol/L ammonium metatungstate × 0.30 mol/L iron (II) chloride × 5 A/m^2^). Refer to Figure 9.

### 3.5. Cycle Time

**Figure 10:**
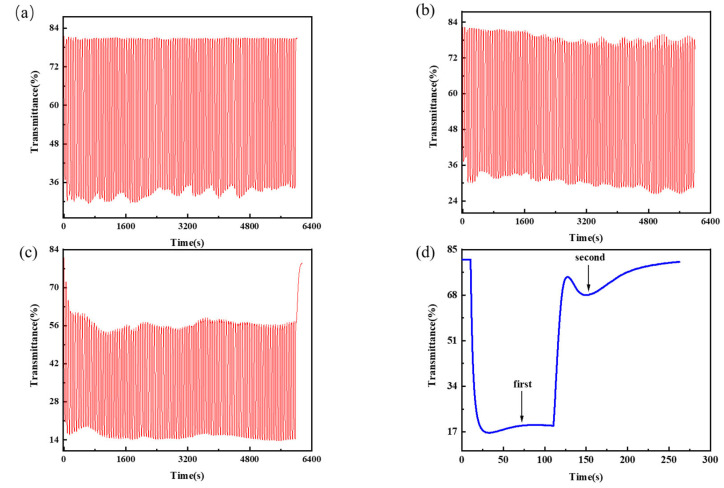
Time diagram of transmittance for 100 cycles of ammonium metatungstate concentration × iron (II) chloride concentration at a current density of 5 A/m^2^: (**a**) 0.175 mol/L × 0.30 mol/L, (**b**) 0.200 mol/L × 0.30 mol/L, and (**c**) 0.225 mol/L × 0.30 mol/L; (**d**) the transmittance time diagram for ammonium metatungstate concentration of 0.225 mol/L and iron (II) chloride concentration of 0.30 mol/L at a current density of 5 A/m^2^.

The performance of the initial ΔT and other parameters of the mixed functional layer was considerably lowered by high voltage, which induced the crystallization of W^4+^ in the solution, which could not be oxidized by the Fe^3+^ in the solution, based on the data collected before. This is proportional to the concentrations of ammonium metatungstate and iron (II) chloride. Thus, the concentrations of 0.175 mol/L ammonium metatungstate × 0.30 mol/L iron (II) chloride, 0.200 mol/L ammonium metatungstate × 0.30 mol/L iron (II) chloride, and 0.225 mol/L ammonium metatungstate × 0.30 mol/L of iron (II) chloride were used as experimental groups for testing the device lifetime. Increasing the concentration to 0.225 mol/L at a current density of 5 A/m^2^, the device life was tested by cycling 100 groups with the 30 s of power on and 30 s of power off. In Figure 10a,b, it can be seen that the electrochromic performance of the devices in these two groups was well maintained during the cycles, always greater than 45%, and the response time was faster. In addition, the fading contrast was less than 10% in 100 cycles, and the 0.175 mol/L × 0.30 mol/L iron (II) chloride group performed better. Although Figure 10c,d show that the 0.225 mol/L ammonium metatungstate × 0.30 mol/L iron (II) chloride group outperformed the measured group by 62%, it was unable to rebound to the cycle’s initial transmittance in the cycle. This phenomenon greatly reduced ∆T in the 0.225 mol/L ammonium metatungstate × 0.30 mol/L iron (II) chloride group in the cycle to less than 40%. Concerning the cause of this phenomenon, the response time increases as the ammonium metatungstate concentration increases, while the group with an ammonium metatungstate concentration of 0.225 mol/L and an iron (II) chloride concentration of 0.30 mol/L cannot return to the original point after energization stops, and the transmittance even decreases, as shown in Figure 10d.

To understand the relationship between capacitance and electrochromism, it is assumed that the Fe^2+^/Fe^3+^ redox pair in the electrolyte acts as an energy store. When W^4+^ is re-oxidized, Fe^2+^ in the solution provides electrons to W^5+^ to reduce it and is converted to Fe^3+^, a reaction that delays the return of the layer to its initial state. When the Fe^2+^ concentration in the solution is high enough, it causes the mixed functional layer to color again. After charging, this mechanism is similar to a capacitor releasing its stored current. This process is inherently weak and hardly affects the electrochromic properties of the mixed functional layer. However, when the concentration of Fe^2+^ in the solution rises, this effect becomes more pronounced, eventually having a significant impact on the electrochromic reaction.

Usually, the size of a capacitor can be determined as follows:

(10) $$C=\frac{\varepsilon S}{4\pi kd}$$

where *ε* is the dielectric constant, *S* is the squared area of the capacitor pole plate, *d* is the distance of the capacitor pole plate, and *k* is the electrostatic force constant.

Generally, the complex dielectric constant can be calculated by the following equation.

(11) $$\varepsilon r\prime \prime =\frac{d}{RS2\pi f\varepsilon 0}$$

where *d* is the sample thickness, *C* is the capacitance, *S* is the electrode plate area, *ε*0 is the vacuum dielectric constant (8.85 × 10^−12^ F m^−1^), *R* is the resistance, and *f* is the frequency of the electric field [47]. Combining (7) and (8) reveals that the resistance of the electrolyte is related to the ions in the solution. Therefore, according to (7), (8) and (11), the capacitance of the device in this experiment is related to the concentrations of the ammonium metatungstate and iron (II) chloride. When the concentration of ammonium metatungstate increases, the capacitance of the device is reduced.

Combining (6) and (7) reveals that the capacitance of the device in this experiment is positively related to the resistance of the electrolyte.

The relationship between capacitance and charge and voltage can be derived from the following equation:

(12) $$C=\frac{Q}{U}$$

where *C* is the capacitance, *Q* is the total amount of charge, and *U* is the voltage. Macroscopically, as the concentration of ammonium metatungstate increases, the resistance per unit volume of the solution decreases, leading to a decrease in capacitance, and from (10), it can be deduced that *U* = “*Q*”/”*C*”. During the fading process of the experimental group containing 0.225 mol/L ammonium metatungstate and 0.30 mol/L iron (II) chloride, *Q* increases while C decreases, increasing *U*. The discharge can be viewed as another electrochromic discoloration since the device cannot distinguish between positive and negative electrodes, which is why the second valley in Figure 10d emerges (More content about this principle can be found in the Supplementary Materials). Meanwhile, the Fe^2+^/^3+^ redox pair also contributes to the electrochromism [48]. Therefore, when the concentrations of [H_2_W_12_O_40_]^6+^ and Fe^2+^/Fe^3+^ in the solution are too high, they still have an effect on the response time and the electrochromic effect.

## 4. Conclusions

The performance of an electrochromic device based on ammonium metatungstate–iron (II) chloride electrochromic liquid is investigated in this paper. One can obtain the optimal device settings by altering the ammonium metatungstate concentration (0.175 mol/L), current density (5 A/m^2^), and the concentration of iron (II) chloride (0.30 mol/L). In addition, the analysis showed an excellent ΔT (57% at 700 nm), response time (about 8.5 s), and coloring efficiency (160.04 cm^2^/C). The discoloration phenomenon of the device at high current densities was found, and the mechanisms and principles were explored in depth. The theory of secondary electrochromic effects due to capacitor discharge is proposed, which may provide ideas and a basis for subsequent research.

## Figures and Tables

**Table 1:** The 25 groups under the Taguchi design.

Code Number	Ammonium Metatungstate (mol/L)	Current Density (A/m^2^)	Iron (II) Chloride (mol/L)
**#1**	0.100	1	0.10
**#2**	0.100	2	0.15
**#3**	0.100	3	0.20
**#4**	0.100	4	0.25
**#5**	0.100	5	0.30
**#6**	0.125	1	0.15
**#7**	0.125	2	0.20
**#8**	0.125	3	0.25
**#9**	0.125	4	0.30
**#10**	0.125	5	0.10
**#11**	0.150	1	0.20
**#12**	0.150	2	0.25
**#13**	0.150	3	0.30
**#14**	0.150	4	0.10
**#15**	0.150	5	0.15
**#16**	0.175	1	0.25
**#17**	0.175	2	0.30
**#18**	0.175	3	0.10
**#19**	0.175	4	0.15
**#20**	0.175	5	0.20
**#21**	0.200	1	0.30
**#22**	0.200	2	0.10
**#23**	0.200	3	0.15
**#24**	0.200	4	0.20
**#25**	0.200	5	0.25

**Table 2:** Comparison of electrochromic (EC) materials, film preparation methods, and transmittance modulation (ΔT) between the literature and this work.

Materials	Film Preparation Method	ΔT (%)	Reference
**Ammonium Metatungstate–Iron (II) Chloride**	None—film	57% (700 nm)	This work
**WO_3_**	RF magnetron sputtering	57% (633 nm)	[37]
**WO_3_/PEDOT**	Spray coating	48% (633 nm)	[38]
**WO_3_**	DC magnetron sputtering	57% (550 nm)	[39]

**Table 3:** Comparison of electrochromic (EC) materials, film preparation methods, and response time (t_c_, t_b_) between the literature and this work.

Materials	Film Preparation Method	Response Time(s) t_b/_t_c_	Reference
**Ammonium Metatungstate–iron (II) chloride**	None—film	8.5/6	This work
**m-WO_3-x_ nanowires**	Spin coating	16/13	[40]
**WO_3_**	Magnetron sputtering	4.0/7.1	[41]
**WO_3_ (nanocrystals** **embedded in the amorphous matrix)** **Ammonium** **metatungstate (ethylene glycol and ammonium** **hydroxide)**	Electron beam evaporation Inkjet print	12/12 2.8/10.4	[42] [27]

**Table 4:** Comparison of electrochromic (EC) materials, film preparation methods, and coloring efficiency (CE) between the literature and this work.

Materials	Film Preparation Method	CE (cm^2^/C)	Reference
**Ammonium** **metatungstate–iron (II) chloride**	/	160.04 cm^2^/C	This work
**WO_3_**	RF magnetron sputtering	27.7 cm^2^/C	[37]
**WO_3_**	Electrodeposition	51 cm^2^/C	[23]
**WO_3_**	Reactive-gas-flow sputtering	35 cm^2^/C	[44]
**WO_3_ (amorphous)** **WO_3_·2H_2_O (nanosheets)**	Sputtering Filtration	72 cm^2^/C 120.9 cm^2^/C	[45] [46]

## Data Availability

Not applicable.

## Supplementary Materials

The following supporting information can be downloaded at: https://www.mdpi.com/article/10.3390/mi13081345/s1, Figure S1: The absorbance diagrams and transmittance diagrams of different solution samples; Figure S2: The voltage-time diagram of different samples of the experiment; Figure S3: The voltage-time cycle diagram of different samples of the experiment.
