# Supplementary material for: Investigation of an Electrochromic Device Based on Ammonium Metatungstate-Iron (II) Chloride Electrochromic Liquid
Source: Micromachines (Basel). 2022 Aug 19;13(8):1345. doi: 10.3390/mi13081345 (PMC9460927; doi:10.3390/mi13081345)
Supplement: Supplementary file 1 [file micromachines-13-01345-s001.zip › micromachines-1850567-supplementary .pdf]

Supplementary Materials

We test all solution samples by UV-VIS, which can clearly show the absorbance at 550 nm decrease with decreasing ferrous chloride concentration. The color at 550 nm is the color of our solution samples. Besides, through the Lambert-Beer law (1), we can get the transmittance by the basic data. The result of it reflects that our device's initial state and bleach state will be influenced by the ferrous chloride concentration when the samples' ammonium metatungstate concentrations are the same. Because the cuvette is too thick for the light to penetrate. In addition, the solution is highly concentrated and absorbs too strongly. So, we don't discuss this in the text.

$$A = -\lg(T) \tag{S1}$$

Where A is the absorbance, T is the transmittance.

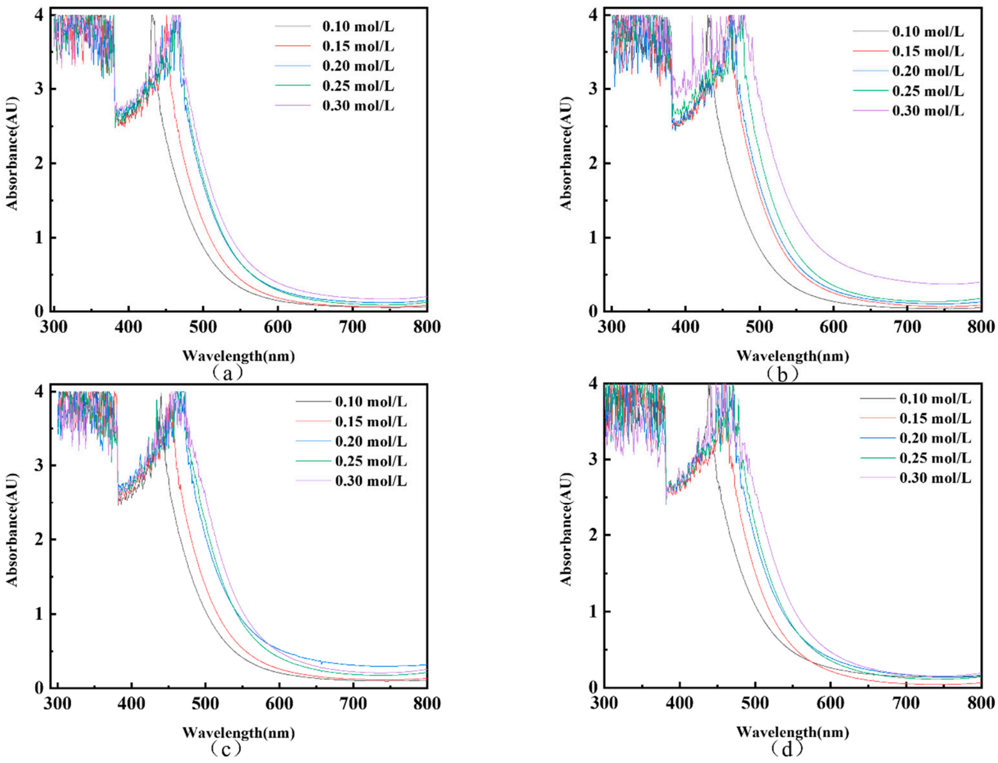

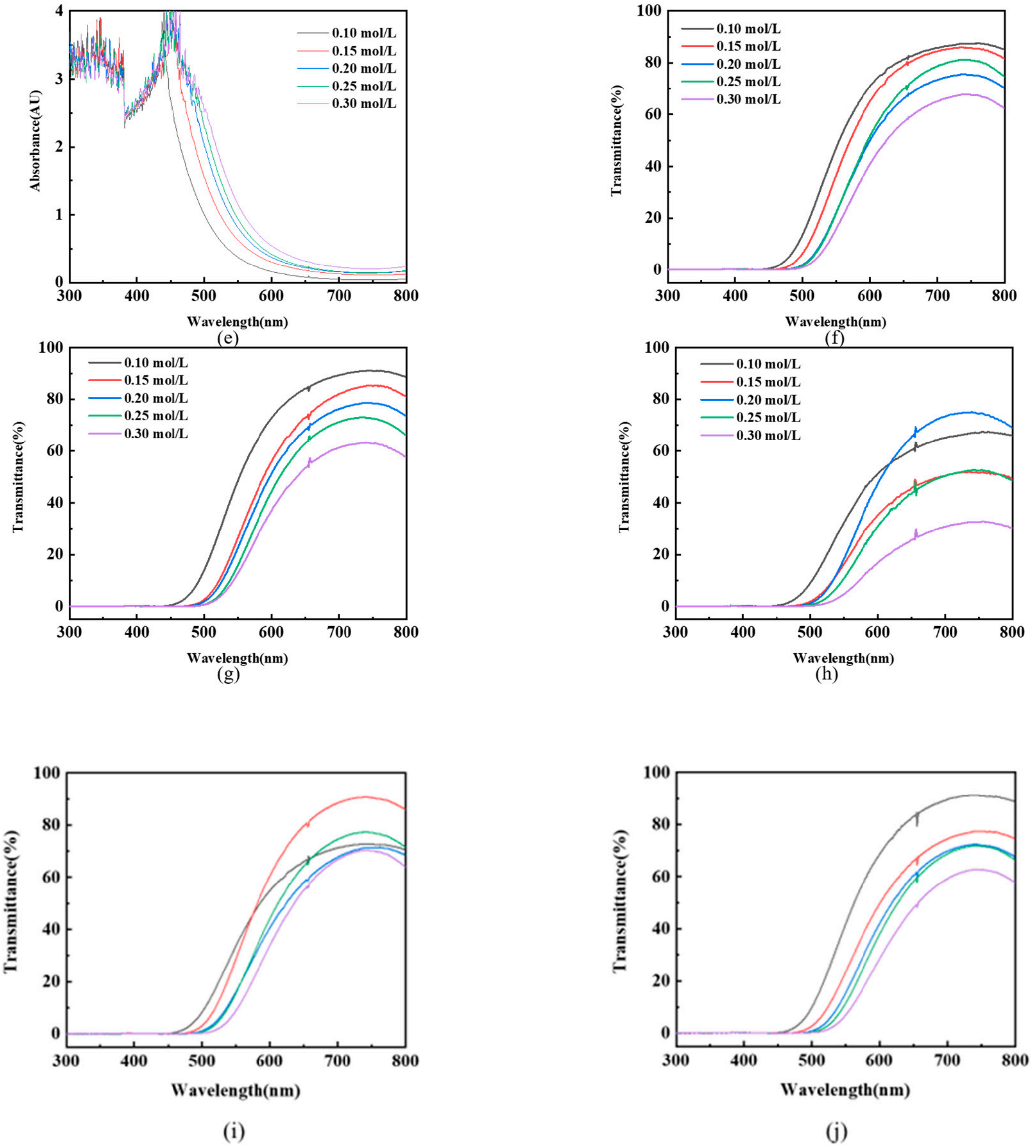

Figure S1. The absorbance diagrams and transmittance diagrams of different solution samples: (**a**) the absorbance diagrams 0.100 mol/L ammonium metatungstate with five levels of iron (II) chloride; (**b**) the absorbance diagrams 0.125 mol/L ammonium metatungstate with five levels of iron (II) chloride; (**c**) the absorbance diagrams 0.150 mol/L ammonium metatungstate with five levels of iron (II) chloride; (**d**) the absorbance diagrams 0.175 mol/L ammonium metatungstate with five levels of iron (II) chloride; (**e**) the absorbance diagrams 0.200 mol/L ammonium metatungstate with five levels of iron (II) chloride; (**f**) the absorbance diagrams 0.100 mol/L ammonium metatungstate with five levels of iron (II) chloride; (**g**) the transmittance diagrams 0.125 mol/L ammonium metatungstate with five levels of iron (II) chloride; (**h**) the transmittance diagrams 0.150 mol/L ammonium metatungstate with five levels of iron (II) chloride (**i**) the transmittance diagrams 0.175 mol/L ammonium metatungstate with five levels of iron (II) chloride; (**j**) the transmittance diagrams 0.200 mol/L ammonium metatungstate with five levels of iron (II) chloride.

We have obtained five equations, which are based on 5 level of current density. Considering the association of research, we only put the equation based on highest level of current density in the text. We hope to add other equations in the supplement materials.

Current density:

1 A/m^2 $\Delta T = -0.84 + 67.2\ C_A + 13.6\ C_F$

2 A/m^2 $\Delta T = 15.56 + 67.2\ C_A + 13.6\ C_F$

3 A/m^2 $\Delta T = 17.76 + 67.2\ C_A + 13.6\ C_F$

4 A/m² $\Delta T = 23.36 + 67.2\ C_A + 13.6\ C_F$

5 A/m² $\Delta T = 29.76 + 67.2\ C_A + 13.6\ C_F$

From Figure S2, the connection between the concentration of ammonium metatungstate and resistance can be researched deeply. Comparing Figure S2a,c can show that keeping the current density and iron (II) chloride level the same, the higher concentration of ammonium metatungstate command lower voltage. Because of the constant current approach, same level of current density, and Ohm's Law, the sample of Figure S2c owns lower resistance. The gap between Figure S2a,b is more obvious, the sample of Figure S2b's voltage lower than a, under the level of higher current density and the same level of iron (II) chloride. Hence, the samples of Figure S2b's resistance is much lower than the samples of Figure S2a. So, we can obtain the primary principle that the high concentration of ammonium metatungstate owns lower resistance. Meanwhile, Figure S2c,d show that the sample of Figure S2c owns a lower voltage than Figure S2d, under the same level of concentration of ammonium metatungstate and current density with different concentrations of iron (II) chloride. Hence, the principle about the concentration of iron (II) chloride and resistance is the same as the principle of ammonium metatungstate. As a result of the aforementioned derivation and the text, the resistance decreases with the concentration of ammonium metatungstate and ferrous chloride in the solution increases.

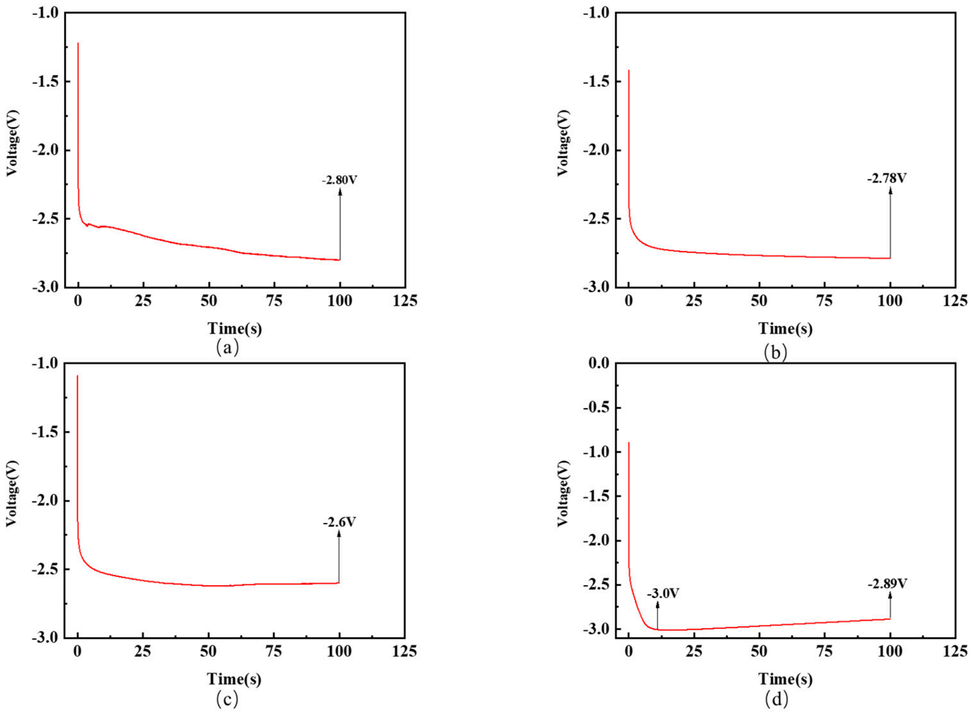

Figure S2. The voltage-time diagram of different samples of the experiment: (**a**) the voltage-time diagram of 0.100 mol/L ammonium metatungstate × 0.30 mol/L iron (II) chloride with 5 A/m²; (**b**)the voltage-time diagram of 0.175 mol/L ammonium metatungstate × 0.30 mol/L iron (II) chloride with 6 A/m²; (**c**) the voltage-time diagram of 0.125 mol/L ammonium metatungstate × 0.30 mol/L iron (II) chloride with 5 A/m²; (**d**) the voltage-time diagram of 0.125 mol/L ammonium metatungstate × 0.10 mol/L iron (II) chloride with 5 A/m².

From Figure S3, we can get obvious diagrams of capacitor charging and discharging. Figure S3b,d show that its voltage can't back to 0 V. So, their transmittance-cycle diagrams, Figure 10b,c can't show the transmittance back to the initial state because the voltage's existence keeps the electrochromic progress. Moreover, Figure S3d's voltage continues higher than 0.5 V, so Figure 10c reflect the worst ΔT during the cycle. Meanwhile,

when the power is switched off, Figure S3d shows a voltage is higher than Figure S3b, refer to Equations (10)–(12), which can counterpart to the discussion in the text that a higher concentration of the solution will increase the voltage of the device when power is switched off and capacitor discharge.

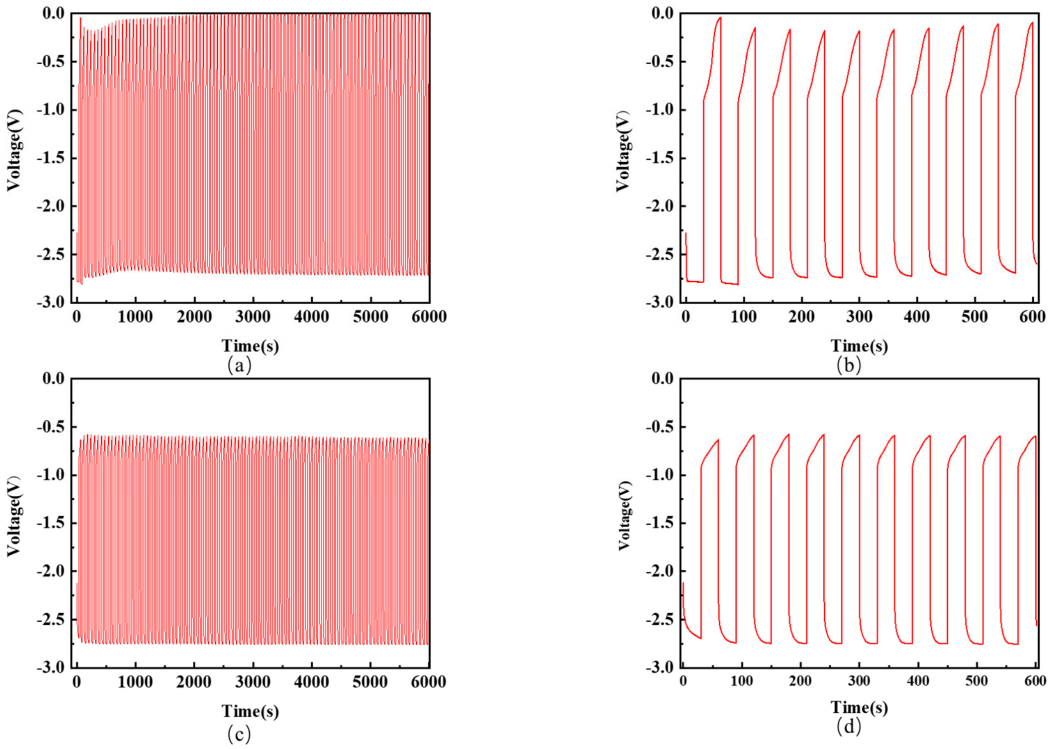

Figure S3. The voltage-time cycle diagram of different samples of the experiment: (**a**) the complete diagram of 0.200 mol/L ammonium metatungstate × 0.30 mol/L iron (II) chloride with 5 A/m^2; (**b**) the initial 10 cycle diagram of 0.200 mol/L ammonium metatungstate × 0.30 mol/L iron (II) chloride with 5 A/m^2; (**c**) the complete diagram of 0.225 mol/L ammonium metatungstate × 0.30 mol/L iron (II) chloride with 5 A/m^2; (**d**) the initial 10 cycle diagram of 0.225 mol/L ammonium metatungstate × 0.30 mol/L iron (II) chloride with 5 A/m^2.
